# Three-dimensional MRI shows cartilage defect extension with no separation from the meniscus in women in their 70 s with knee osteoarthritis

**DOI:** 10.1038/s41598-022-08092-5

**Published:** 2022-03-10

**Authors:** Hisako Katano, Nobutake Ozeki, Hideyuki Koga, Makoto Tomita, Kenji Suzuki, Jun Masumoto, Ichiro Sekiya

**Affiliations:** 1 Center for Stem Cell and Regenerative Medicine, Tokyo Medical and Dental University, 1-5-45 Yushima, Bunkyo-ku, Tokyo, 113-8510 Japan; 2 Department of Joint Surgery and Sports Medicine, Graduate School of Medical and Dental Sciences, Tokyo Medical and Dental University, Tokyo, Japan; 3 School of Data Science, Graduate School of Data Science, Yokohama City University, Yokohama, Japan; 4 Fujifilm Corporation, Tokyo, Japan

**Keywords:** Stem cells, Medical research

## Abstract

The positional relationship between cartilage defects and the meniscus is poorly understood for osteoarthritis of the knee. Our purpose was to clarify how cartilage defects extend and their association with the meniscus location during osteoarthritis progression. The subjects were women in their 70 s who were registered in the Kanagawa Knee Study. We obtained 3D MRI images of the tibial surfaces with menisci in subjects with cartilage area ratios < 0.95 and examined the morphological association between cartilage defects and the medial meniscus (MM) by viewing the defects according to the cartilage area ratio at the medial tibial region. Of the 561 Kanagawa Knee Study subjects, 45 were included in the analyses, and 11 had a cartilage area ratio < 0.95 at the medial tibia. Significant differences were observed in the localization of cartilage defects among 9 subregions, with cartilage defects occurring predominantly in the middle external subregion. The inner margin of the MM contacted the cartilage defect in 7 knees and crossed the cartilage defect in 4 knees but was never found separated from the cartilage defect. The cartilage defects occurred from the middle external subregion and extended to the surrounding area without separating from the inner margin of the MM.

*Trial registration* UMIN, UMIN000032826; 1 September 2018.

## Introduction

Osteoarthritis (OA) of the knee is a degenerative disease characterized by cartilage wear and spur formation^1^. An increasing number of papers now report that the main cause of medial OA, which accounts for the majority of knee OA, is extrusion of the medial meniscus (MM)^2^. Cadaveric studies have shown that MM extrusion (MME) concentrates the load distribution on the articular cartilage^3^, while animal studies have shown that MME accelerates articular cartilage wear^4^. However, how MME affects the morphology of articular cartilage in humans has not been established.

Morphological studies have previously examined the association between the medial tibial cartilage regions and MM. For example, almost 80 years ago, Bennet described that cartilage damage was most prevalent in the tibial area not covered by the meniscus^5^. Gulati et al. reported that the midpoint of the medial tibial cartilage defect was, on average, 3.4% anterior to the compartment midline in patients undergoing unicompartmental knee arthroplasty for OA^6^. Arno et al.^7^ analyzed the bone pieces resected during total knee arthroplasty for OA and reported a medial tibial cartilage defect located central in an anterior–posterior direction and extending to the medial margin. Arno et al.^8^ examined OA knees by magnetic resonance imaging (MRI) and reported an inverse relationship between the medial tibial cartilage volume and the meniscus contact ratio (the proportion of MM length to the medial femoral cartilage length in one MRI slice). Although cadaveric observations and surgical and 2D MRI studies have been reported, no account has yet been taken of the 3D structure of the cartilage and meniscus under conditions where the soft tissue is not damaged. Knowledge is also lacking regarding the morphological relationship between the medial tibial cartilage and the MM. Clarification of this association could lead to improvements in the prevention and treatment of OA.

Recent advances in analysis systems have opened up the possibility of automatically extracting the cartilage and meniscus from knee MRI information and using 3D images to obtain a noninvasive determination of the morphological relationship between the cartilage and meniscus^9^. However, using this image extraction method to clarify the progression of knee OA requires periodic observations of MRI images of specific subjects, beginning before the onset of OA and progressing through the late stages of OA. This necessitates data collection over a considerable period and is difficult to accomplish. One approach to solve this problem is to collect cross-sectional MRI data from a relatively homogeneous population, arrange them in order of OA severity from mild to severe by sensitive quantitative evaluation, and then analyze the morphological relationship between the medial tibial cartilage and MM, assuming that OA progresses in the same order.

The Kanagawa Knee Study is our current epidemiological survey, and we have collected MRI data for 3D analysis from 561 current and former desk workers. All the study subjects had no history of hospital visits for knee disease for more than 3 months^9^. Here, we have extracted data from the Kanagawa Knee Study only for female participants aged in their 70 s, as this population theoretically has a relatively well-matched set of risk factors for OA, including age, gender, environment, and history of trauma^10^. The purpose of our current study was to clarify how cartilage defect extension occurs and to determine its association with the meniscus location during OA progression. We therefore viewed 3D MRI images of the knees of this selected cohort who showed medial tibial cartilage defects and arranged them from highest to lowest in terms of the ratio of the remaining cartilage area to the region of interest (ROI) in the medial tibial cartilage. Our hypothesis was that the cartilage defects extend from the inner margin of the extruded MM.

## Methods

### The Kanagawa knee study

This study was approved by the Medical Research Ethics Committee of Tokyo Medical and Dental University, and written informed consent was obtained from all subjects. All methods were performed in accordance with the relevant guidelines and regulations. The protocols were enrolled in a database of the National University Hospital Council of Japan (UMIN000032826) on 01/06/2018 and disclosed. The first subject (registration number: KS-001) was included in the study 28/09/2018. The last subject (registration number: KS-573) was included on 05/09/2019.

The purpose of the Kanagawa Knee Study is to clarify the epidemiology and natural history of knee OA^9^. The main inclusion criteria are (1) employees of the Kanagawa Prefectural Office, retired employees of the Kanagawa Prefectural Office, or those who work in Kanagawa Prefecture or live in the Tokyo metropolitan area, and (2) those who work at a desk for at least 4 h per day or perform similar work during their employment. The main exclusion criteria are those who have (1) a history of surgery on either the left or right knee and (2) a past history of consecutive visits to the hospital for more than 3 months for knee injuries on either the left or right side, from birth to the start of data collection.

### MRI scanning

The MRI system was used at 3.0 T (Achieva 3.0TX, Philips, Amsterdam, Netherlands) with 16-channel coils. The sagittal plane of the knee joint was acquired to obtain both a fat-suppressed spoiled gradient echo sequence (SPGR) image and a proton-density weighted (PDW) image, with total scan durations of 7 min 30 s and 7 min 10 s, respectively. For both images, sagittal images were obtained at an in-plane resolution of 0.31 × 0.31 mm, a partition thickness of 0.36 mm (320 slices), and a field of view (head to tail × anterior to posterior) of 150 × 150 mm^11^.

### MRI 3D images and cartilage area ratio

The MRI analysis software was a 3D image analysis system volume analyzer (SYNAPSE 3D (Japanese product name: SYNAPSE VINCENT), collaborative version, FUJIFILM Corporation, Tokyo, Japan). The cartilage regions were automatically extracted from the SPGR images, and the bone and meniscus regions were obtained from the PDW images. The reconstructed 3D images were projected onto a plane. The cartilage thickness mapping was also displayed.

**Figure 1:**
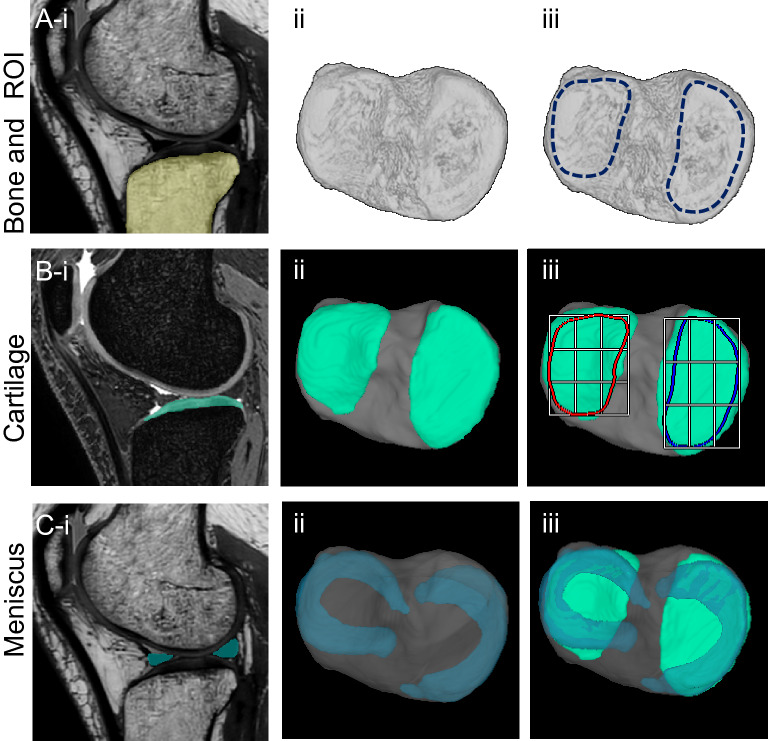
3D MRI analysis of the tibial surface of a representative control knee. (**A**) Bone and ROIs. The PDW images were used for automatic segmentation of the bone region (yellow) (**i**), the 3D image was then reconstructed (**ii**), and the medial and lateral tibial articular surfaces were defined as the ROIs (**iii**). (In this image, the ROIs were drawn manually for explanatory purposes.) (**B**) Cartilage. The SPGR images were used for automatic segmentation of the cartilage region (green) (**i**), the tibial articular cartilage and bone were reconstructed in 3D (**ii**), and ROIs with nine subregions were overlapped (**iii**). (**C**) Meniscus. The PDW images were used for automatic segmentation of the meniscus regions (blue) (**i**), the medial meniscus and lateral meniscus were reconstructed in 3D and overlapped on the bone (**ii**), and the cartilage was inserted (**iii**).

The software set the ROI at the medial tibial surface according to the bone morphology (Fig. 1, Supplementary Fig. 1). The software automatically computed a “cartilage area ratio,” which represented the ratio of the cartilage area to the total area of the ROI. A cartilage area ratio value of 1 indicates that cartilage covers the entire ROI, whereas a cartilage area ratio value of 0 means that no cartilage covers the ROI. The software drew a bounding box around the ROI, that was partitioned equally into three vertical and horizontal subregions, generating thus nine subregions^9^.

We previously validated our software and determined the Dice similarity coefficients (DSC), an index of accuracy, between the software-generated contours and the manually generated contours^12^. The average DSC was 0.98 for the tibial bone, 0.89 for the tibial cartilage, 0.92 for the MM, and 0.89 for the ROI of the medial/lateral tibia plateau^9^.

### Radiographic images

Anteroposterior radiographic images were obtained with the knee in extension in the standing position. The medial compartment was classified by Kellgren-Lawrence (KL) grading^13^.

### Medial meniscus extrusion distance

The MRI digital imaging and communications in medicine (DICOM) data were also analyzed using SYNAPSE 3D software. Coronal cross-sectional images were reconstructed from MRI data of proton-enhanced sagittal cross-sectional images. The slice in which the tibia was at its maximum transverse diameter was selected. The meniscus area was automatically extracted, and a perpendicular line was manually drawn from the inner edge of the tibia, excluding the spurs. A second perpendicular line was also manually drawn from the outer edge of the MM. The distance between these two perpendicular lines was defined as the MME distance^14^.

### Enrollment of subjects

**Figure 2:**
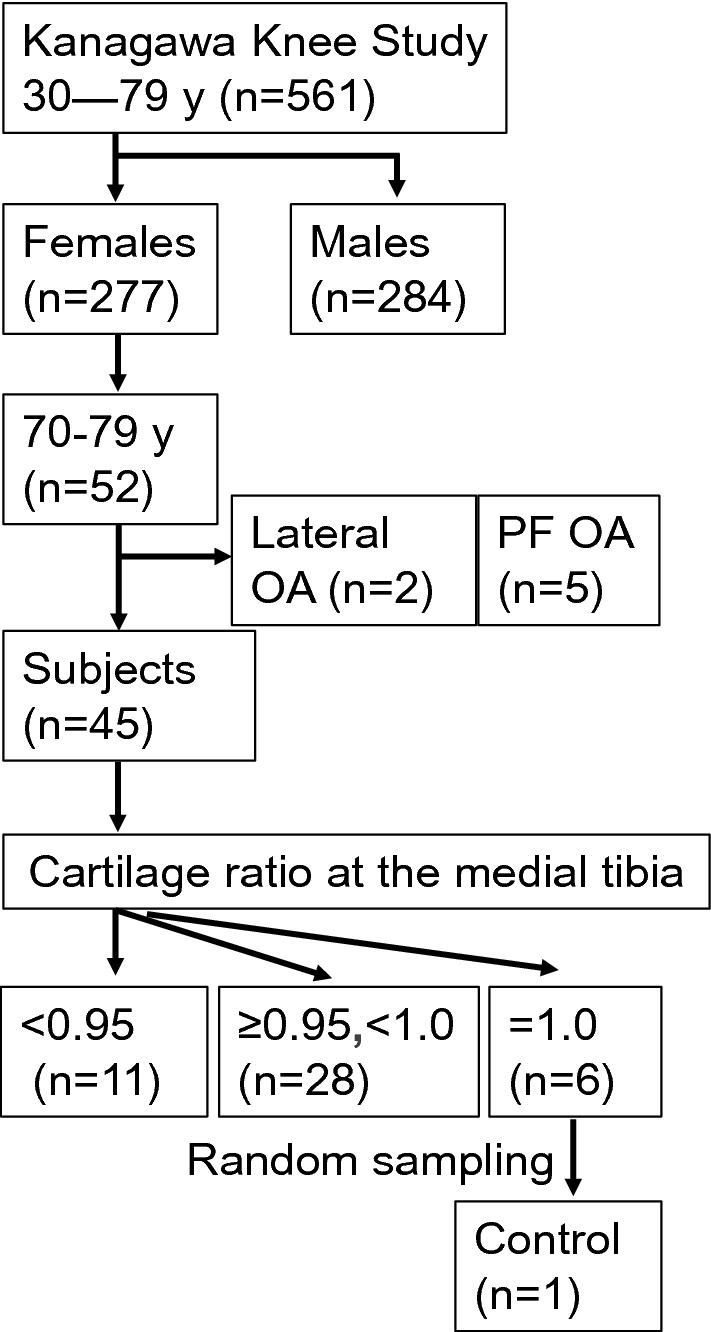
Enrollment of subjects. The Kanagawa Knee Study consisted of 561 subjects, including 277 females, 52 of whom were women 70–79 years old. Excluding 2 subjects with lateral osteoarthritis (OA) and 5 subjects with patellofemoral (PF) OA, 45 subjects were selected for analysis in this study; 11 of these subjects had cartilage area ratios at the medial tibial < 0.95. One control subject was randomly selected from 6 subjects with cartilage area ratios of 1.0.

The Kanagawa Knee Study included 561 subjects. Of these, 277 were females, and 52 of those women were between the ages of 70 and 79 (Fig. 2). After excluding 2 subjects with lateral OA and 5 subjects with patellofemoral (PF) OA, 45 subjects were selected for analysis in the present study. Of these, 11 subjects had cartilage ratios < 0.95 at the medial tibia. One control subject was also randomly selected from 6 subjects with a cartilage area ratio of 1.0.

### Statistical analysis

The association between the cartilage area ratio and the KL grade was examined in 45 subjects using the Jonckheere-Terpstra test. The MME distance was examined using the Welch two-sample t-test to determine the significance of any difference between “the cartilage area ratio < 0.95” group (n = 11) and “the cartilage area ratio ≥ 0.95” group (n = 34). Pearson's Chi-square test was used in 11 subjects with cartilage ratio < 0.95 to examine whether cartilage defects occur equally in the nine subregions at the medial tibial cartilage, with the assumption that the null hypothesis was equally valid in all nine subregions.

The relationship between the inner margin of the MM and the cartilage defect can be classified into three categories: contact, intersection, and separation. We examined if these three categories occurred equally using Pearson's Chi-squared test and again assuming that the null hypothesis was equally valid in all three categories. A value of *p* < 0.05 was considered statistically significant. All statistical analyses were performed with EZR software (Saitama Medical Center, Jichi Medical University, Saitama, Japan)^15^.

### Ethics approval and consent to participate

This study was approved by the Medical Research Ethics Committee of Tokyo Medical and Dental University, and written informed consent was obtained from all subjects. The protocol was enrolled in a database of the National University Hospital Council of Japan (UMIN000032826) and disclosed.

### Consent for publication

This manuscript does not contain any identifiable information about an individual.

## Results

### Radiographic images

**Figure 3:**
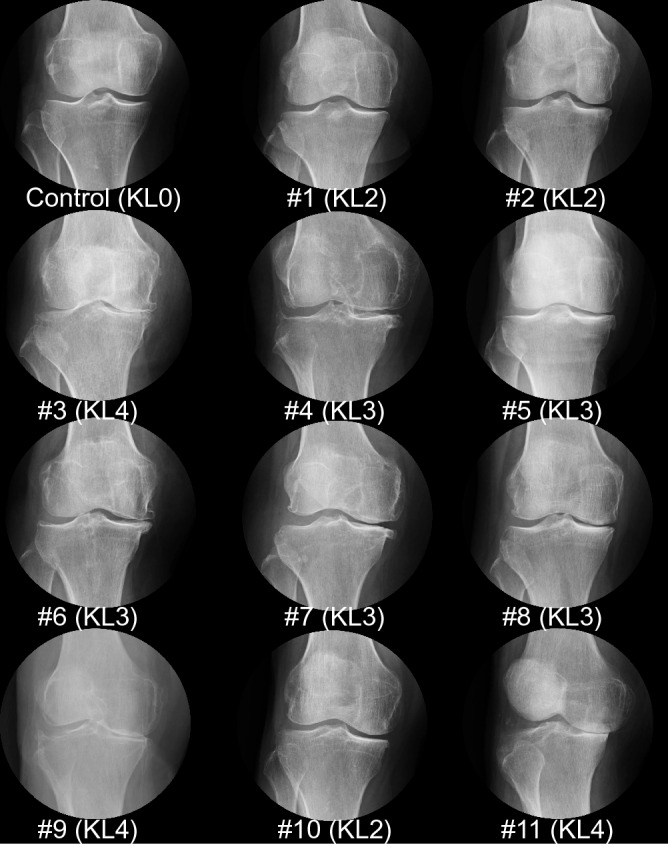
Anteroposterior radiographic images of the knee in the standing position. The images are ordered from highest to lowest in terms of their cartilage area ratios at the medial tibia. The Kellgren–Lawrence grade is also shown.

Of the 34 subjects with a cartilage area ratio ≥ 0.95, 26 had KL0-1 and 8 had KL2 according to anteroposterior radiographic images of the knee in the standing position. By contrast, of the 11 subjects with a cartilage area ratio < 0.95, 3 had KL2, 5 had KL3, and 3 had KL4 (Fig. 3). No significant relationship was evident between the cartilage area ratio and the KL grade in the subjects with a cartilage area ratio < 0.95.

### Medial meniscus extrusion by 2D MRI

**Figure 4:**
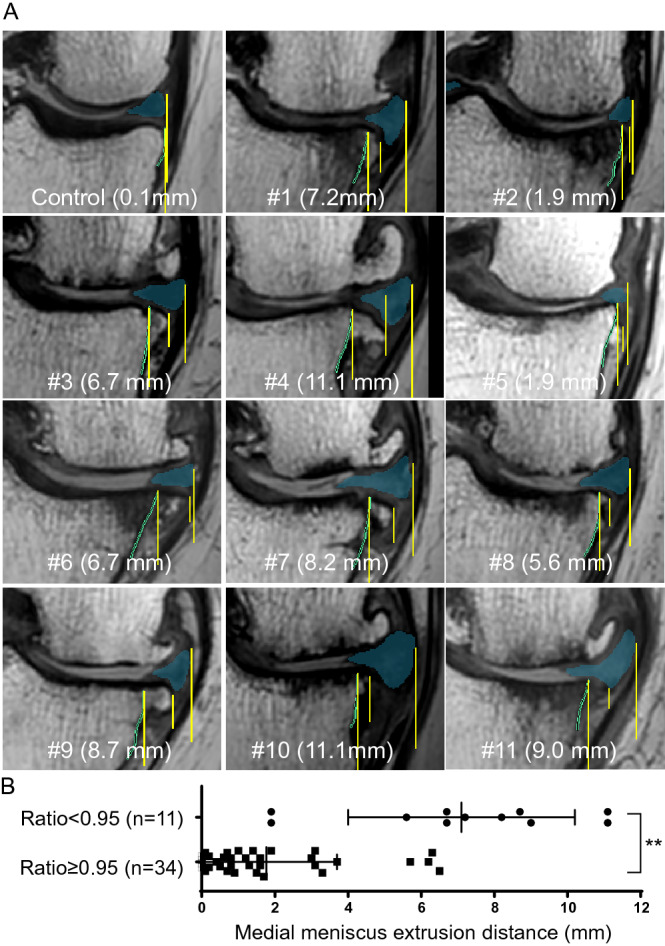
Medial meniscus (MM) extrusion determined by 2D MRI. (**A**) Medial meniscus in coronal images of proton-weighted MRI. The images are ordered from highest to lowest cartilage area ratios at the medial tibia; the MM extrusion distance is also shown. The three yellow lines indicate, from the center, the vertical line of the outer margin of the tibia, the vertical line of the outer margin of the spur, and the vertical line of the outer margin of the MM. (**B**) Comparison of the MM extrusion distance in the two groups separated by cartilage area ratios at the medial tibia of 0.95. Average with SD is shown. **; *p* < 0.01 by the Welch two-sample t-test.

The control subject showed no tibial spur in 2D MRI (Fig. 4A). By contrast, 11 subjects with a cartilage area ratio < 0.95 showed a variety of spurs at the medial tibia. The MME was 1.8 ± 1.9 mm (standard ± SD; n = 34) in the subjects with a ratio ≥ 0.95 and 7.1 ± 3.1 mm (n = 11) in the subjects with a ratio < 0.95 and this MME difference was statistically significant (Fig. 4B).

### 3D MRI of the cartilage

**Figure 5:**
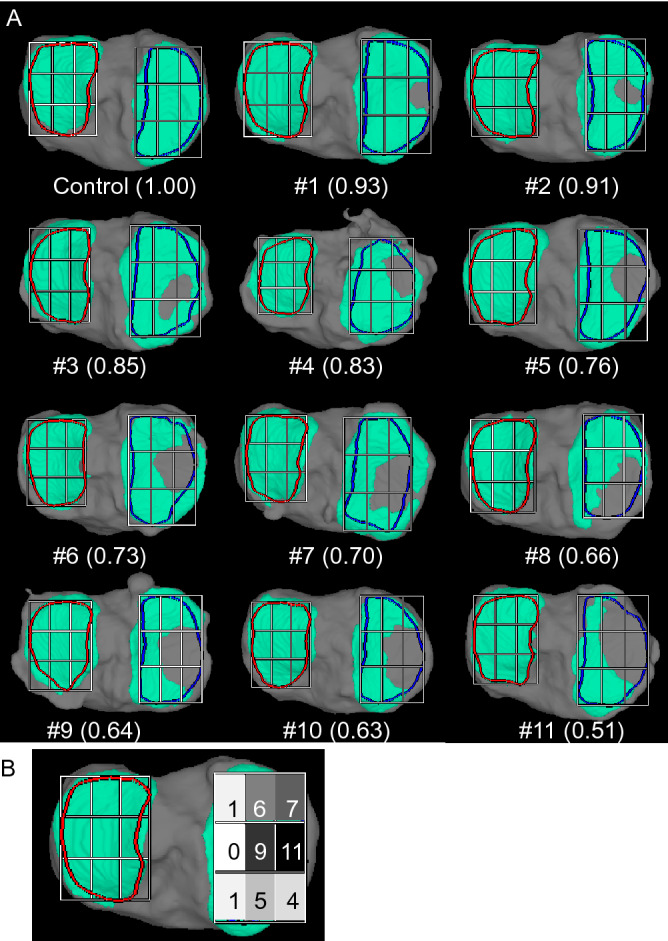
Location of defects in the medial tibial cartilage. (**A**) Cartilage area of the tibia determined by 3D magnetic resonance imaging (MRI). The tibial cartilage is shown in green, and the region of interest (ROI) of the medial tibial cartilage is shown enclosed within the blue line in the fully automated 3D-constructed MRI image. The cartilage area ratio is defined as the ratio of the cartilage area to the ROI area. The cartilage area ratio at the medial tibia is also shown, and the images are ordered from the highest to the lowest ratios. The ROI of the medial tibial cartilage was divided into 9 subregions, with 3 equal vertical and 3 equal horizontal lines. (**B**) Number of knees with cartilage defects in each subregion among 11 knees. The thickness of the cartilage is indicated by color mapping, where white indicates a thickness ≥ 2 mm and the progression from yellow to red indicates thinning of the cartilage. The tibia bone is shown in gray.

In all 11 knees with cartilage defects, the defects were located in the middle (with respect to the anterior–posterior axis) external (with respect to the internal–external axis) subregion of the nine subregions (Fig. 5A). In particular, cartilage defects were prevalent in the five subregions adjacent to the middle external subregion (Fig. 5B), and the difference in localization was statistically significant.

### Superimposition of the meniscus on the cartilage thickness map by 3D MRI

**Figure 6:**
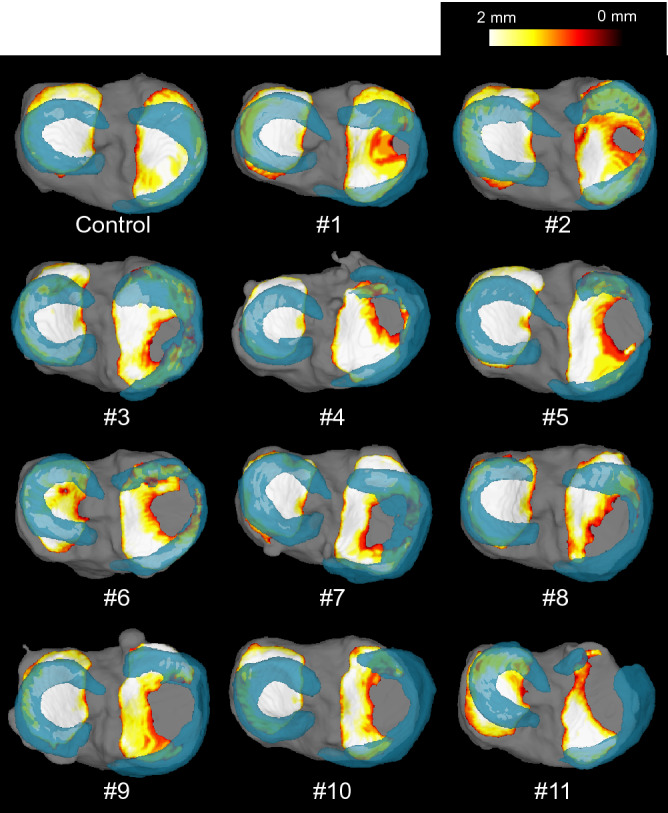
Superimposition of the meniscus on the cartilage thickness mapping of the tibia by 3D MRI. The thickness of the cartilage is indicated by color mapping, where white indicates a thickness ≥ 2 mm and the progression from yellow to red indicates thinning of the cartilage. The meniscus is shown in translucent blue, and the tibia bone is shown in gray.

**Figure 7:**
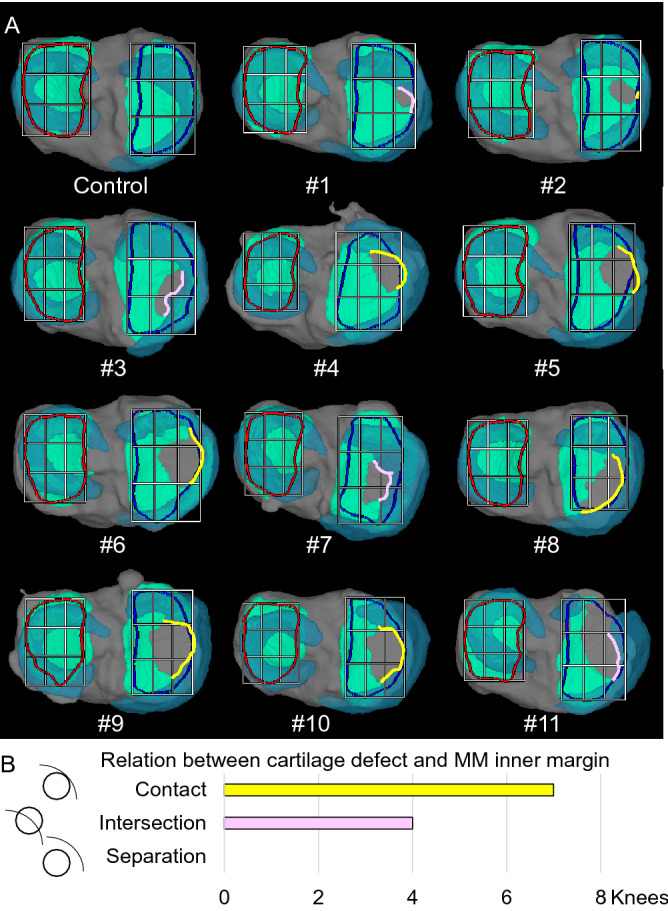
Relationship between cartilage defects and the inner margin of the medial meniscus (MM). (**A**) 3D MRI images of the articular surface of the tibia. The tibial cartilage is shown in green, the meniscus in translucent blue, and the bone in gray. The line where the inner margin of the MM contacts the cartilage defect is shown in yellow, and the line where the inner margin of the MM crosses the cartilage defect is shown in pink. (**B**) Quantitative evaluation of the relationship between cartilage defects and the inner margin of the MM.

The 3D MRI images showed the anatomical location of the MM in the control (Fig. 6). The inner margin contacted the cartilage defect in 7 OA knees (67%) and crossed the cartilage defect in 4 OA knees (33%), but was never separated from the cartilage defect in any knees (0%) (Fig. 7A). The number of knees in which the inner margin of the MM was separated from the cartilage defect was significantly lower than in the other categories (Fig. 7B).

## Discussion

We viewed 3D MRIs of knees with medial tibial cartilage defects in order of severity in women in their 70 s to determine how the cartilage defects expanded and how they were related to the location of the meniscus during the progression of OA. The cartilage defects were located in the middle external subregion in all 11 knees with cartilage defects. These findings indicated that the cartilage defects were initiated in the middle external subregion and extended to the surrounding subregions.

We included 45 women in their 70 s from the 561 subjects in the Kanagawa Knee Study, after excluding those with lateral OA and PF OA. Twenty of the 45 subjects (44%) had scores of KL2 or higher. This proportion was lower than that reported in the ROAD Study, a large OA study in Japanese subjects, in which 72% of the 913 women in their 70 s had scores of KL2 or higher^16^. This difference probably reflects: (1) our participant restriction to subjects who were (or had been) engaged in desk work; (2) our exclusion of subjects who had been hospitalized for knee disease for more than 3 months; (3) our exclusion of subjects with lateral OA and PF OA; and (4) our inclusion of subjects who mainly lived in urban areas.

This study excluded 7 participants with lateral OA and PF OA from the 52 female subjects in their 70 s because OA in those 7 women obviously differed from medial OA in its pathogenesis^6^. The major factor in medial OA is the varus alignment, whereas it is valgus alignment in lateral OA^17^ and abnormally patellar tilt or congruence in PF OA^18^.

“Morphological analysis of the 3D MRI excluded 34 participants with a cartilage area ratio greater than 0.95 and less than 1.00. Three orthopedic surgeons (NO, HKo, and IS), who specialize in knee surgery and have more than 20 years of experience, looked at the 3D MRI of the 34 knees. They attributed these readings to the setting of the ROI rather than to cartilage defects due to the aging process (Supplementary Fig. 2). We also assume that the cartilage defects with a cartilage area ratio of less than 0.95 in the 3D MRI are similar to those generally observed during total knee arthroplasty for knee OA. Therefore, we considered further analysis of the subjects with a cartilage area ratio less than 0.95 to be worthwhile in this study. Since the DSC of the ROI of the medial/lateral tibia plateau is 0.89, rather than 1.00^9^, the ROI extends outside the cartilage area in some cases, which is one of the limitations of this software.

Only one of the six participants with a cartilage area ratio = 1 was randomly selected as a control. This participant was presented as a representative reference case for presenting 3D MRI with a cartilage area ratio of less than 0.95. We did not treat this one participant as an exceptional subject in the statistical analysis.

No significant relationship was evident between the cartilage area ratio and the KL grade in the subjects with a cartilage area ratio less than 0.95. This is a result of the small sample size. We recently reported that the radiographic minimum joint space width at the medial compartment was correlated with the cartilage thickness in 3D MRI analysis and that the radiographic osteophyte width was correlated with the MME distance in the 3D MRI analysis in the subjects aged 30–79 years old in the Kanagawa Knee Study^19^. Analysis of this dataset (n = 527) demonstrated that the correlation coefficient between the cartilage area ratio and the radiographic minimum joint space width at the medial compartment was 0.28 (*p* = 4.0 × 10–11), indicating a weak correlation^20^.The correlation coefficient between the cartilage area ratio and the radiographic medial tibial osteophyte width was -0.42 (*p* = 4.6 × 10–24), indicating a moderate correlation.

The MME was significantly greater in the subjects with a cartilage area ratio < 0.95 at the medial tibial region than in the subjects with a ratio ≥ 0.95. Our study supports the many previous reports on the relationship between medial knee OA and MME^21–23^. Identifying the boundary of the outer edge of the MM is sometimes difficult in practice using 2D MRI, but this operation was easy in this study because the MM was automatically extracted and colored beforehand. This study is the first to show an association between the MME distance and the cartilage area ratio that is automatically calculated by 3D MRI analysis.

The 3D MRI scans of the cartilage and the MM demonstrated that the inner margin of the MM contacted or intersected the cartilage defect, but no cases were observed where the two were separated. This means that the cartilage defect extended without separating from the MM in OA of the knee. The inner margin of the MM intersected the cartilage defect in 4 of the 11 knees; however, since MM extrusion progresses more in the standing position than in the supine position, performing the MRI in the standing position instead of the supine position would cause the medial margin of the MM to come into contact with the cartilage defect in these knees. Kawaguchi et al. measured MME by ultrasonography and reported increases in the MME distance in the standing position compared to the supine position of 0.48 mm in KL2, 1.06 mm in KL3, and 0.42 mm in KL4 in females^24^.

We speculate that the progression of OA, based on the findings from this study and previous reports, occurs as follows: extrusion of the MM, which is an important cause of OA, causes the medial femoral condyle to come into direct contact with the medial tibial cartilage that lacks the MM. Extrusion of the MM also induces varus alignment^25^ and/or lateral thrust^26^. These changes result in cartilage defects and further enlargement of those defects.

Our study had three limitations. One was that spurs could not be determined automatically by 2D MRI. In addition, the localization of the native cartilage was ambiguous in 3D MRI because spurs and chondrophytes could not be indicated. A second limitation was the subjective nature of the evaluation of the association between the cartilage defect area and the inner margin of MM. A more objective quantitative analysis of this positional relationship is needed for further implementation of our protocol. The third limitation is that detailed meniscus conditions, such as meniscus injuries, meniscus root tears, and meniscus degeneration, are not shown. A more detailed elucidation of how meniscus pathology affects MME and, consequently, the cartilage will be important for elucidating the mechanism of OA progression.

## Conclusion

Knees with cartilage defects were complicated by a high rate of MM extrusion. The cartilage defects were initiated at the middle external subregion and extended to the surrounding area without separating from the inner margin of the MM.

## Supplementary Information

Supplementary Figures.

Supplementary Figure Legends.

## Data Availability

The data sets used and/or analyzed during the current study are available from the corresponding author on reasonable request.

## Abbreviations

MRI: Magnetic resonance imaging
3D MRI: Three-dimensional MRI
2D MRI: Two-dimensional MRI
OA: Osteoarthritis
MM: Medial meniscus
MME: MM extrusion
PF OA: Patellofemoral OA
SPGR: Spoiled gradient
PDW: Proton-density weighted
KL: Kellgren–Lawrence
DICOM: Digital imaging and communications in medicine
