# Supplementary material for: Three-dimensional MRI shows cartilage defect extension with no separation from the meniscus in women in their 70 s with knee osteoarthritis
Source: Sci Rep. 2022 Mar 10;12:4198. doi: 10.1038/s41598-022-08092-5 (PMC8913674; doi:10.1038/s41598-022-08092-5)
Supplement: Supplementary file 1 — Supplementary Figures. [file 41598_2022_8092_MOESM1_ESM.pptx]

## Slide 1
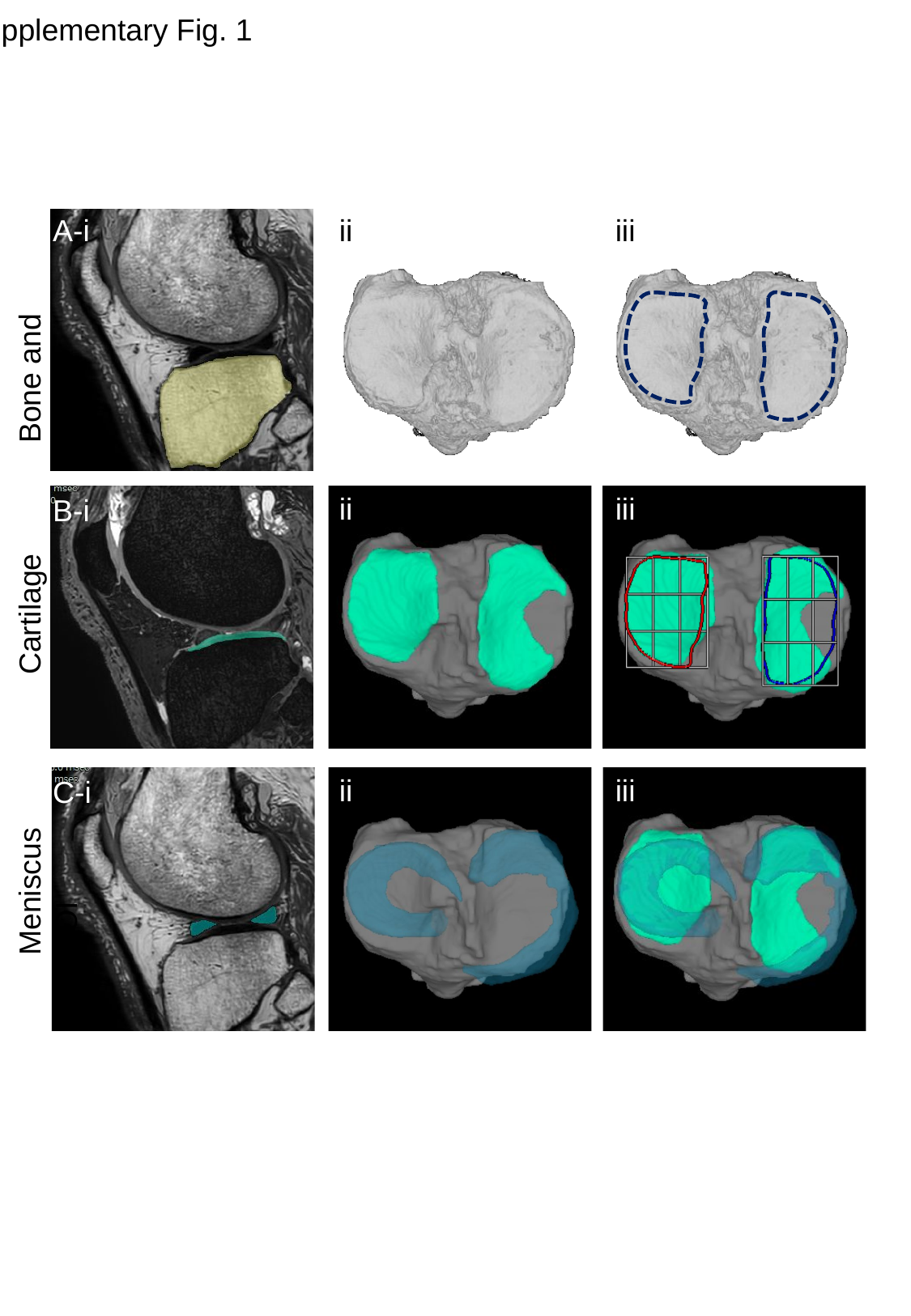

Supplementary Fig. 1
A-i
ii
iii
ii
iii
B-i
Meniscus Cartilage Bone and　ROI
ii
iii
C-i

## Slide 2
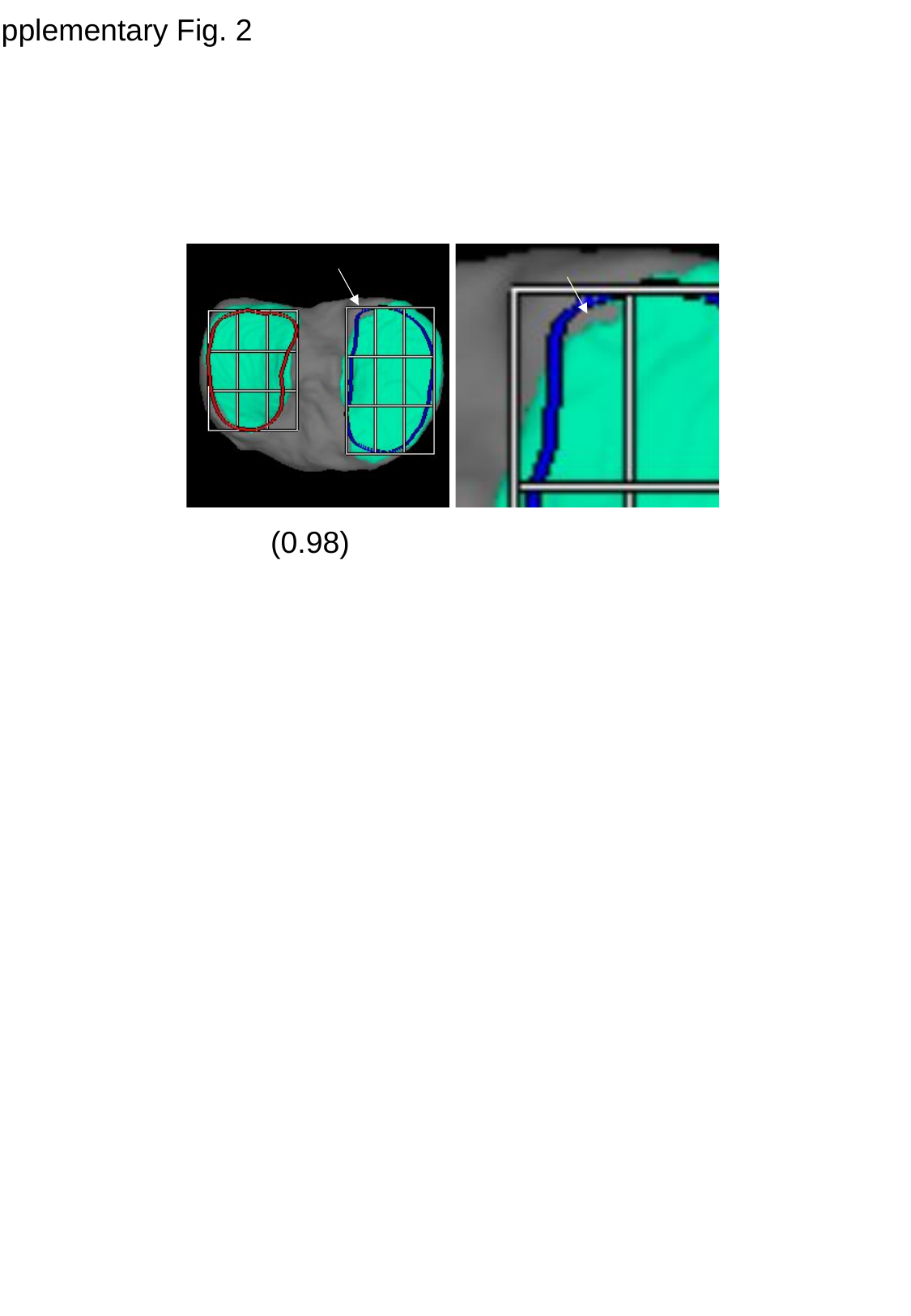

Supplementary Fig. 2
 (0.98)
