# Supplementary material for: Three-dimensional MRI shows cartilage defect extension with no separation from the meniscus in women in their 70 s with knee osteoarthritis
Source: Sci Rep. 2022 Mar 10;12:4198. doi: 10.1038/s41598-022-08092-5 (PMC8913674; doi:10.1038/s41598-022-08092-5)
Supplement: Supplementary file 2 — Supplementary Figure Legends. [file 41598_2022_8092_MOESM2_ESM.docx]

**Supplementary Figure 1.**

**3D MRI analysis of the tibial surface of a representative OA knee.** (A) Bone and ROIs. The PDW images were used for automatic segmentation of the bone region (yellow) (i), the 3D image was reconstructed (ii), and the medial and lateral tibial articular surfaces were defined as the ROIs. (In this image, the ROIs were drawn manually for explanatory purposes.) (B) Cartilage. The SPGR images were used for automatic segmentation of the cartilage region (green) (i), the tibial articular cartilage and bone were reconstructed in 3D (ii), and the ROIs with nine subregions were overlapped (iii). (C) Meniscus. The PDW images were used for automatic segmentation of the meniscus regions (blue) (i), the medial meniscus and lateral meniscus were reconstructed in 3D and overlapped on the bone (ii), and the cartilage was inserted (iii).

**Supplementary Figure 2.**

**Cartilage area and ROIs of a representative knee** with a cartilage area ratio at the medial tibia higher than 0.95 and lower than 1.00. In this particular case, the cartilage area ratio was 0.98. The cartilage area in the ROI is strangely lacking (arrow), possibly due to a mismatch between the ROI and the cartilage area.
